# Evaluation of K18-*hACE2* Mice as a Model of SARS-CoV-2 Infection

**DOI:** 10.4269/ajtmh.20-0762

**Published:** 2020-07-28

**Authors:** Gregory Brett Moreau, Stacey L. Burgess, Jeffrey M. Sturek, Alexandra N. Donlan, William A. Petri, Barbara J. Mann

**Affiliations:** 1 Division of Infectious Diseases and International Health, Department of Medicine, University of Virginia School of Medicine, Charlottesville, Virginia;; 2 Division of Pulmonary and Critical Care Medicine, Department of Medicine, University of Virginia School of Medicine, Charlottesville, Virginia;; 3 Department of Microbiology, Immunology, and Cancer Biology, University of Virginia School of Medicine, Charlottesville, Virginia;; 4 Department of Pathology, University of Virginia School of Medicine, Charlottesville, Virginia

## Abstract

Murine models of SARS-CoV-2 infection are critical for elucidating the biological pathways underlying COVID-19. Because human angiotensin-converting enzyme 2 (ACE2) is the receptor for SARS-CoV-2, mice expressing the human *ACE2* gene have shown promise as a potential model for COVID-19. Five mice from the transgenic mouse strain K18-*hACE2* were intranasally inoculated with SARS-CoV-2 Hong Kong/VM20001061/2020. Mice were followed twice daily for 5 days and scored for weight loss and clinical symptoms. Infected mice did not exhibit any signs of infection until day 4, when no other obvious clinical symptoms other than weight loss were observed. By day 5, all infected mice had lost around 10% of their original body weight but exhibited variable clinical symptoms. All infected mice showed high viral titers in the lungs as well as altered lung histology associated with proteinaceous debris in the alveolar space, interstitial inflammatory cell infiltration, and alveolar septal thickening. Overall, these results show that the K18-*hACE2* transgenic background can be used to establish symptomatic SARS-CoV-2 infection and can be a useful mouse model for COVID-19.

## INTRODUCTION

An invaluable step in identifying effective vaccines and therapies to combat COVID-19 is the availability of a mouse model of infection. The host receptor for SARS-CoV-2 is the human angiotensin-converting enzyme 2 (hACE2),^1^ which was previously identified as the receptor for the SARS-CoV-1^2^ that causes SARS, a disease that emerged from China in 2002.^3^ The mouse ACE2 ortholog, which has significant amino acid sequence variation in the viral receptor binding domain, cannot serve as an efficient receptor for either SARS-CoV-2 or CoV-1.^4^ A transgenic mouse model to study SARS-CoV-1 infection was developed that expresses the *hACE2* gene under the control of the human cytokeratin 18 promoter.^5^ Infection of these mice with SARS-CoV-1 results in a rapidly lethal infection.^5^ Four other *hACE2*-expressing mouse lines have been created to date and tested for the ability to support SARS-CoV-2 infection. Two lines express the *hACE2* gene under the control of the mouse *ACE2* promotor^6,7^; one was made using the CRISPR/Cas9 technology.^7^ The third strain uses the lung ciliated epithelial cell hepatocyte nuclear factor-3/forkhead homologue 4 (HFH4) promoter.^8,9^ An additional approach was to transfect wild-type mice with an adenovirus carrying the *hACE2* gene.^10^ Overall, with the exception of the HFH4 mice, in which there was some lethality, infection of these three mouse strains with SARS-CoV-2 results in mild clinical symptoms and no lethality. Here, we report the infection of K18-*hACE2* with SARS-CoV-2. Although this infection resembled that of other strains, we observed variable clinical presentation, with some mice exhibiting more severe symptoms than reported using other models. Overall, this work supports the usefulness of K18-*hACE2* transgenic mice as a model for human COVID-19 infections.

## RESULTS AND DISCUSSION

**Figure 1.**
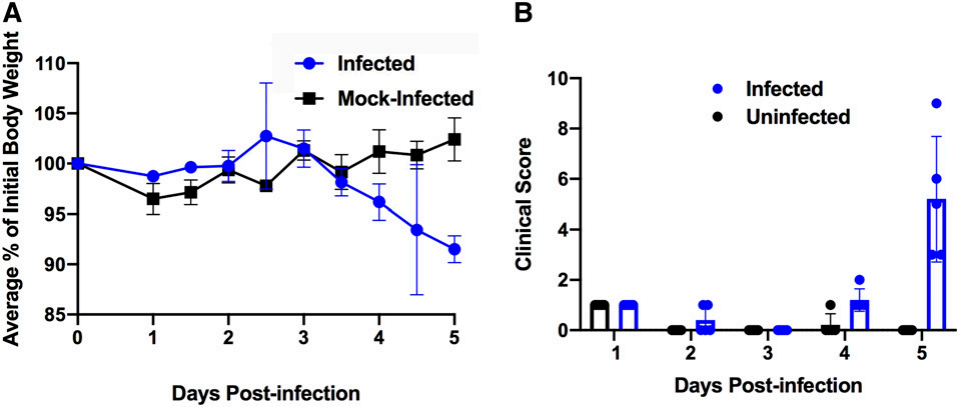
K18*-hACE2* C57Bl/6J mice were intranasally inoculated with 8 × 10^4^ TCID_50_ of SARS-CoV-2, and weight loss and clinical score were monitored. (**A**) Weight loss is measured as the percent weight loss compared with the initial weight on day 0. (**B**) Clinical score consists of weight loss, activity level, eye closure, appearance of fur and posture, and respiration. The mock-infected mice did not exhibit any clinical symptoms or experience any weight loss throughout the experiment.

To investigate the potential of this transgenic mouse strain as a model for COVID-19 infection, five K18-*hACE2* mice were intranasally inoculated with 8 × 10^4^ Median Tissue Culture Infectious Dose (TCID50) of SARS-CoV-2, and five mice were mock-infected with sterile Dulbecco’s Modified Eagle’s Medium (DMEM). Mice were followed twice daily for 5 days and scored for clinical symptoms (weight loss, eye closure, appearance of fur [piloerection] and posture, and respiration). The mock-infected mice did not exhibit any clinical symptoms or experience any weight loss throughout the experiment. Infected mice did not exhibit any measurable clinical symptoms through day 3. On day 4, no other clinical symptoms other than weight loss were observed. On day 5, all the infected mice had lost around 10% of their original weight (Figure 1A) and exhibited variability in other clinical signs of infection, with clinical scores ranging from 3 to 9 (maximal score 14) (Figure 1B). Although two of the infected K18-*hACE2* mice showed only mild symptoms at day 5 (weight loss and reduced activity), two mice exhibited piloerection. The most severe mouse had increased respiration, lethargy, and slight eye closure and met our criteria for euthanasia. Because the study was ended on day 5, it is unclear whether the remaining four mice would have recovered if the study was carried past day 5.

Although the clinical severity was variable between infected K18-*hACE2* mice, our results suggest that these mice present with more symptomatic disease than other *hACE2* mouse models of SARS-CoV-2 infection. In the mouse model expressing *hACE2* under the mouse *ACE2* promoter, infected mice did not exhibit any clinical symptoms other than maximal weight loss on day 3 postinfection, and those mice recovered.^7^ Only mild ruffling of fur and up to 8% weight loss on day 5 were observed in the other model using the mouse *ACE2* promoter, and once again, all mice recovered.^6^ In mice transfected with an adenovirus carrying the *hACE2* gene, mice exhibited about a 10% weight loss on day 4 postinfection but no lethality.^10^ In contrast to these models, in which mice exhibited mild symptoms and recovered, only 60% of the mice survived past day 5 in the mouse strain expressing *hACE2* under the lung ciliated epithelial cell *HFH4* promoter.^9^ Although this model had higher lethality, weight loss was only about 5% and these mice had no respiratory symptoms. The authors hypothesize that mortality may be due to neuroinvasion because virus was detected in the brain. In K18-*hACE2* mice infected with SARS-CoV-1, the course of infection is clearly different; the infection is uniformly fatal, beginning on day 4 postinfection, and mice were symptomatic with labored breathing and lethargy.^5^ Although the number of mice used in this study was small and we were not able to measure survival, our data support a difference in the disease progression between these two viruses.

**Figure 2.**
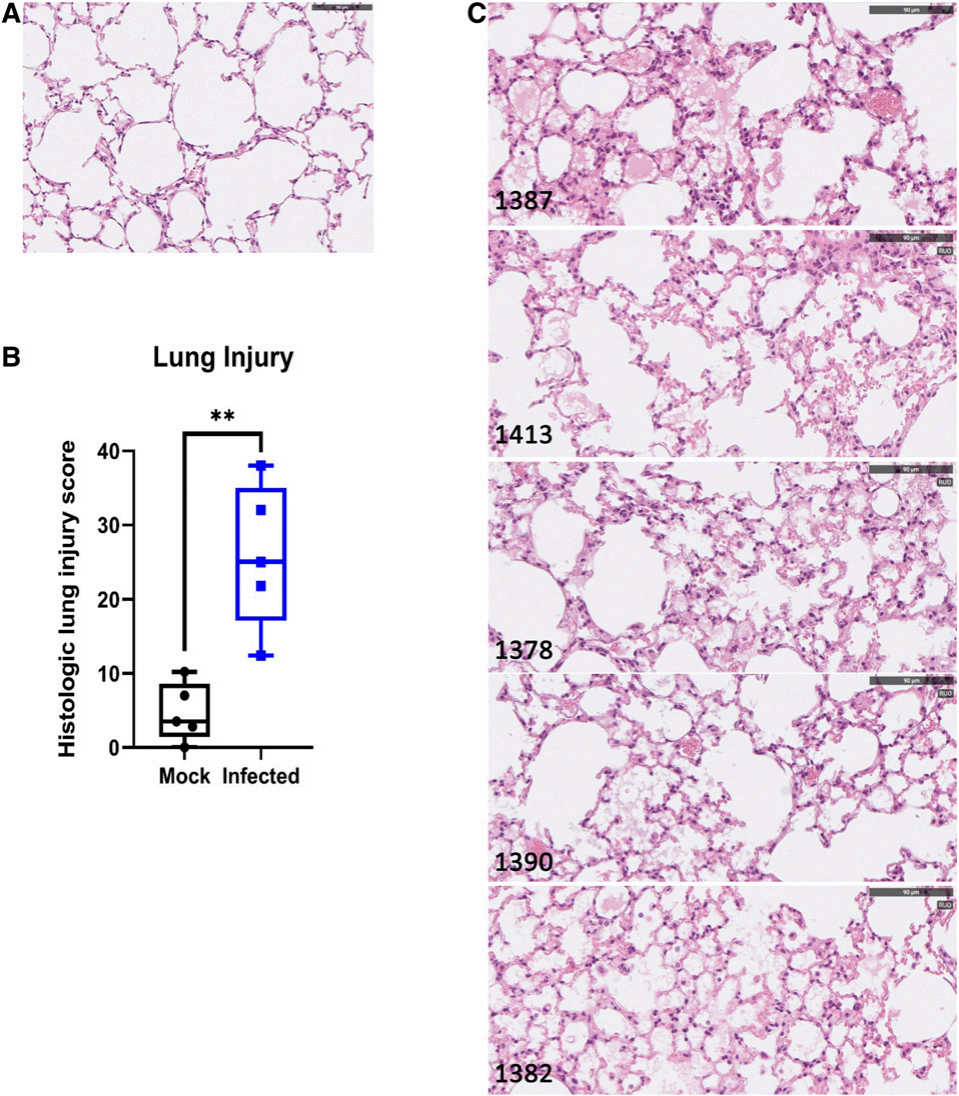
Lung histology of mice infected with SARS-CoV-2. Representative images from hematoxylin and eosin stains of from lungs of infected mice (**A**) and mock-infected (**B**) mice. Lungs from infected mice had alveolar proteinaceous debris, interstitial inflammatory cell infiltration, and alveolar septal thickening. Blinded quantification of lung injury is shown in **C**. Scale bar = 90 µm. **P < 0.01. A two-tailed Student’s *t* test was used to determine statistical significance.

**Figure 3.**
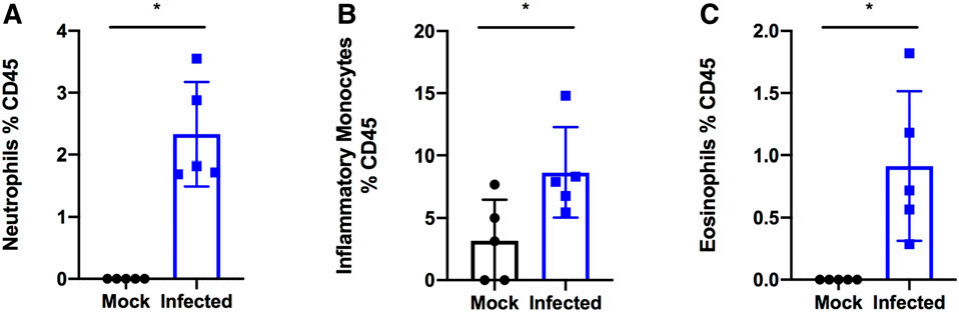
SARS-COV-2 infection increases granulocytes and inflammatory monocytes in the bronchoalveolar lavage fluid. K18-hACE2 C57Bl/6J mice were intranasally inoculated with 8 × 10^4^ TCID_50_ of SARS-CoV-2 Hong Kong/VM20001061/2020 (Source: BEI Resources). Bronchoalveolar lavage was collected, cells isolated, and stained via flow cytometry. *P < 0.05. A two-tailed Student’s *t* test was used to determine statistical significance.

All mice were euthanized on day 5, and tissue was collected for dissection and enumeration of viral loads. No significant differences in histology of the spleen, small intestine, or liver were observed between infected and mock-infected mice, and these tissues were normal in size and appearance. Dissection of the lungs of infected mice revealed a mottled or marbled appearance that was not observed in mock-infected mice (data not shown). Lung sections were analyzed after staining with hematoxylin and eosin and scored based on tissue pathology.^11^ SARS-CoV-2–infected mice exhibited significantly higher histopathology scores than mock-infected mice (Figure 2). The major histopathology findings in infected mice were proteinaceous debris in the alveolar space, neutrophils in the interstitial space, and alveolar septal thickening (Figure 2); these observations were consistent with other h*ACE2* mouse models, which also detected signs of lung injury including interstitial pneumonia, inflammatory cell infiltrates, and alveolar septal thickening.^6,7^ Consistent with the observed infiltrating neutrophils, granulocytes and inflammatory monocytes were also elevated in the bronchoalveolar lavage (BAL) fluid from the infected mice (Figure 3).

Other *hACE2* mouse models of COVID-19 infection have observed high viral titers in the lungs with limited viral load in organs such as the liver and spleen during intranasal infection.^6,7^ Although we did not investigate viral load in the liver or spleen, these organs appeared normal by histology, suggesting that there was limited viral titer in these tissues. Virus was detected in the lungs of all infected mice, with titers generally in the range of 1 × 10^5^ plaque forming units (PFU)/mL (Table 1). Viral titers in the lungs appeared somewhat associated with disease severity: mouse 1390, which had the highest lung titer, had the highest clinical score, histopathology score, and percent weight loss at day 5 (Table 1) and the highest numbers of neutrophils, monocytes, and eosinophils in the BAL (Figure 3). In addition, mouse 1413, which had the lowest titer, had the lowest clinical score, second lowest percent weight loss at day 5 (Table 1), and lowest number of eosinophils and monocytes in the BAL (Figure 3). Of note, mouse 1413 did not have the lowest histopathology score (Table 1). Although there were trends toward higher viral titers in the lungs being associated with higher clinical and histopathology scores, these trends were not significant, and viral titer was not a strong predictor of percent weight loss. The power of this analysis is limited by the small sample size, but these results suggest that factors in addition to viral load, such as inflammatory responses, are driving the severity of disease. This would also potentially explain the sudden onset of clinical symptoms at 5 days post-inoculation.

**Table 1:** Characteristics of infected mice on day 5 postinfection

Mouse ID	% Of initial weight (day 5)	Clinical score	Average lung pathology score	Viral load in lungs (PFU/mL)
1413	91.6	3	24	7.5 × 10^3^
1378	91.2	3	32	1.2 × 10^5^
1390	89.7	9	38	1.75 × 10^5^
1387	91.6	5	12.4	5.0 × 10^4^
1382	93.5	6	21.8	1.5 × 10^5^

In this report, we have described the course of SARS-CoV-2 infection in K18-*hACE2* transgenic mice. Our findings are consistent with other studies using *hACE2* mice, which observed successful infection with SARS-CoV-2 and a milder disease severity compared with SARS-CoV-1.^6,7^ The onset of symptoms was abrupt, manifesting on day 5. Mice exhibited a similar degree of weight loss but a varying degree of symptoms and clinical/histopathological scores. The number of mice used in this study was too small to determine whether this was a result of experimental variability or natural variability in outcomes. The variance in clinical and histopathological scores may be partially explained by viral titer, but there are likely other factors, such as the host immune response, that contribute to the variance observed. The observation of more severe disease in a subset of the K18-*hACE2* mice is distinct from other *hACE2*-expressing COVID-19 models, which typically observed only mild clinical symptoms.^6,7^ This could be due to experimental differences such as strain differences or the challenge dose (Table 2). To date, little is known about the possibility of virulence differences among isolates. Hong Kong/VM20001061/2020 and strain 2019n-CoV/USA_WA1/2020 are closely related and have been classified as Type IB.^12^ The receptor-binding domains of these strains are 100% identical (data not shown). The phylogeny of HB-01 and Wuhan/AMMS01/2020 has not been reported. The challenge dose used in each experiment is similar; our experiment used the lowest amount of inoculum. The resident microbiota in each mouse strain could also impact outcomes of infection. The other difference between these strains is in the level of *hACE2* receptor expression or tissue distribution. Nonetheless, K18-hACE2 transgenic mice may be a particularly useful for studying the biological processes underlying the clinical symptoms of COVID-19.

**Table 2:** Comparison of *hACE2* mouse challenge outcomes

Expression of *hACE2* gene	Inoculum	Strain	Outcome
K18 promoter (this study)	8 × 10^4^ TCID_50_	Hong Kong/VM20001061/2020	Mild to severe clinical scores, lung pathology present, 10% weight loss
Mouse *ACE2* promotor^6^	10^5^ TCID_50_	HB-01	Mild fur ruffling, 8% weight loss, lung pathology present, all recovered
Mouse *ACE2* promotor (CRISPR/Cas9)^7^	4 × 10^5^ PFU	Wuhan/AMMS01/2020	No clinical signs, 10% weight loss in aged mice only, lung pathology present, all recovered
*HFH4* promoter^8,9^	Not specified	Not specified	∼ 5% Weight loss, no clinical symptoms, but only 60% survived
Adenovirus transfection^10^	10^5^ focus-forming units	Strain 2019n-CoV/USA_WA1/2020	10% Maximum weight loss, all recovered

hACE2 = human angiotensin-converting enzyme 2.

## METHODS

### Challenge.

Five 10-week-old male Tg (K18-*hACE2*) 2Prlmn (Jackson Laboratories, Bar Harbor, ME) mice were challenged with 8 × 10^4^ TCID_50_ in 50 μL of Hong Kong/VM20001061/2020 (NR-52282, Biodefense and Emerging Infections Research Resources Repository (BEI Resources), National Institute of Allergy and Infectious Diseases (NIAID), and National Institutes of Health (NIH)), as measured by BEI Resources, by the intranasal route. Five mock-infected female mice received 50 μL DMEM. Mice were followed twice daily for clinical symptoms until day 5. Categories included in clinical scoring included weight loss (0–5), posture and appearance of fur (piloerection) (0–2), activity (0–3), eye closure (0–2), and respiratory rate (0–2). All mouse work was approved by the university’s institutional animal care and use committee, and all procedures were performed in the university’s certified animal biosafety level three laboratory.

### Histology.

Tissues were fixed in formaldehyde. Slides were scanned at ×20 magnification. Histopathological scoring for lung tissues was performed according to the guidelines of the American Thoracic Society.^11^ A two-tailed Student’s *t* test was used to determined statistical significance.

### Viral titers.

The left lobe of the lung was homogenized in 1 mL serum-free DMEM with a disposable tissue grinder. Plaque assays were performed as described.^13^ In brief, Vero C1008, Clone E6 (ATCC CRL-1586) cells grown in DMEM (GIBCO 11995-040) with fetal bovine serum (FBS) were seeded into at a concentration of 2 × 10^5^ cells/well the night before the assay. Serial dilutions were added to the wells. The plate was incubated at 37°C, 5% CO_2_ for 2 hours, shaking the plates every 15 minutes. After 2 hours, the media was replaced with a liquid overlay of DMEM, 2.5% FBS containing 1.2% Avicel PH-101 (Sigma Aldrich, St. Louis, MO) and incubated at 37°C, 5% CO_2_. After 3 days, the overlay was removed, wells were fixed with 10% formaldehyde, and stained with 0.1% crystal violet to visualize plaques. Plaques were counted, and PFUs were calculated according to the following equation: Average # of plaques/dilution factor × volume diluted virus added to the well.

### Bronchoalveolar lavage fluid and flow cytometry.

Bronchoalveolar lavage was performed, and cells were removed via centrifugation. Cells were stained then fixed in IC fixation buffer (eBioscience, 00-8222-49, San Diego, CA) and run and identified via Zombie NIR (Biolegend, 423105, San Diego, CA), CD45, Alexa Fluor 532 (eBioscience, 58-0459-42, San Diego, CA), CD11c, PE-Cy7 (Biolegend, 117317, San Diego, CA), CD11b, BV480 (BD Biosciences, 566117, San Jose, CA), SIGLEC F, AF700 (eBioscience, 56-1702-80, San Diego, CA), Ly-6c, FITC (Biolegend, 128005, San Diego, CA), and Ly-6G, BV650 (Biolegend, 127641, San Diego, CA) on a Cytek Aurora Borealis at the University of Virginia flow cytometry core. Neutrophils are Zombie NIR^−^, CD45^+^, CD11C^−^, CD11B^+^, and Ly-6G^+^; eosinophils are Zombie NIR^−^, CD45^+^, CD11C^−^, CD11B^+^, and Siglec F^+^; inflammatory monocytes are Zombie NIR^−^, CD45^+^, CD11C^−^, CD11B^+^, Ly-6G^−^, and Ly-6C^++^. Data analysis and figure generation were performed in Omiq and Graphpad Prism. A two-tailed Student’s *t* test was used to determined statistical significance.
